# Evaluation of Gartland Classification, Baumann Angle and Anterior Humeral Line in Paediatrics Supracondylar Fractures: An Inter and Intra-Observer Reliability Study

**DOI:** 10.3390/jcm13010167

**Published:** 2023-12-28

**Authors:** Valeria Calogero, Angelo Gabriele Aulisa, Silvia Careri, Giulia Masci, Giuseppe Mastantuoni, Francesco Falciglia, Renato Maria Toniolo

**Affiliations:** 1 U.O.C Traumatology, Bambino Gesù Children’s Hospital, IRCCS, 00165 Rome, Italy; agabriele.aulisa@opbg.net (A.G.A.); silvia.careri@opbg.net (S.C.); giulia.masci@opbg.net (G.M.); giuseppe.mastantuoni@opbg.net (G.M.); francesco.falciglia@opbg.net (F.F.); rmtoniolo@yahoo.it (R.M.T.); 2 Department of Human Sciences, Society and Health, University of Cassino and Southern Lazio, 03043 Cassino, Italy

**Keywords:** supracondylar fractures, Gartland classification, Baumann angle, anterior humeral line, children

## Abstract

Supracondylar fractures of the humerus are frequent paediatric injuries. The aims of this study were to evaluate the applicability and reproducibility of the Gartland and Wilkins classification, the Baumann angle (BA) and the Anterior Humeral Line (AHL). This retrospective monocentric observational study was conducted on 217 patients. Four observers assessed the pre-operative radiographs by applying the Gartland and Wilkins classification and the post-operative X-rays by measuring the BA and AHL. The kappa coefficient (K) and the Cohen’s kappa were used for the reliability of the Gartland classification; the Intraclass Correlation Coefficient (ICC) for that of the BA. The AHL was evaluated in a double manner by using first the K and the Cohen’s kappa and then the ICC. A total of 186 patients were eligible. Inter-observer reliability for the Gartland classification was K = 0.73–0.61 for type III, 0.65–0.61 for type Ia and 0.43–0.26 for type IIb. The Baumann angle mean value in the first data collection was 73.5 ± 6.85 (inter-observer ICC 0.74) and 72.9 ± 6.83 (inter-observer ICC 0.77) for the second data collection; AHL: inter-observer ICC 0.87 for the first evaluation and 0.80 for the second one. Gartland’s classification modified by Wilkins has a high degree of reliability. BA and AHL appear reproducible and reliable.

## 1. Introduction

Supracondylar fractures of the humerus are frequent injuries typical of the immature skeleton. They account for up to 18% of all pediatric fractures and up to 60% of elbow fractures, with an annual incidence of 177.3 per 100,000 [1,2,3,4]. According to some authors, the male population aged between 5 and 7 years is more affected, and the prevalent site is the non-dominant upper limb [5,6]. Other authors stated that there is a sex-independent distribution and that the left upper limb is more involved [7]. There is a seasonal distribution with a peak in the summer months, probably related to the increased physical and sporting activity during this period. 

The supracondylar region in pediatric age is inherently fragile due to variations in microarchitecture. The antero-posterior and lateral metaphyseal diameters are relatively decreased compared to the adult, the spongy bone trabeculae are weaker, the corticals are thinner, and the olecranon fossa and coronoid fossa are wider in the first decade of age; the distal epiphysis of the humerus is still largely cartilaginous; the ligamentous laxity in the elbow in extension typical of pediatric age favors the development of a bending force in the supracondylar area [8]. 

The most common mechanism of fracture is in hyperextension, when the olecranon acts as a fulcrum in the olecranon fossa, usually following an accidental fall with impact on the hand with the elbow extended (97–99% of cases) [9]. With the hand in pronation, the direction of the dislocation is postero-medial; if it is in supination, the dislocation will be postero-lateral, with different involvement of soft parts and vascular-nervous structures. Alternatively, an anteriorly directed force can be developed in a direct trauma or in a fall with a flexed elbow (2–3% of cases). 

The supracondylar humerus fracture may be associated with a forearm fracture, configuring a “floating elbow” in 3.5–5.3% of cases. Peripheral nerve damage occurs in approximately 10–15% of pediatric supracondylar fractures: the anterior interosseous nerve and the median nerve appear to be involved in extension fractures, and more rarely the radial nerve may be damaged; ulnar nerve injuries occur most commonly in flexion fractures and medial epicondyle fractures. Non-palpable arterial pulse is reported in the literature in 6% of Gartland type III and may be due to spasm, extrinsic constriction, incarceration between fracture fragments, thrombosis and tearing of the vessel wall. 

**Figure 1:**
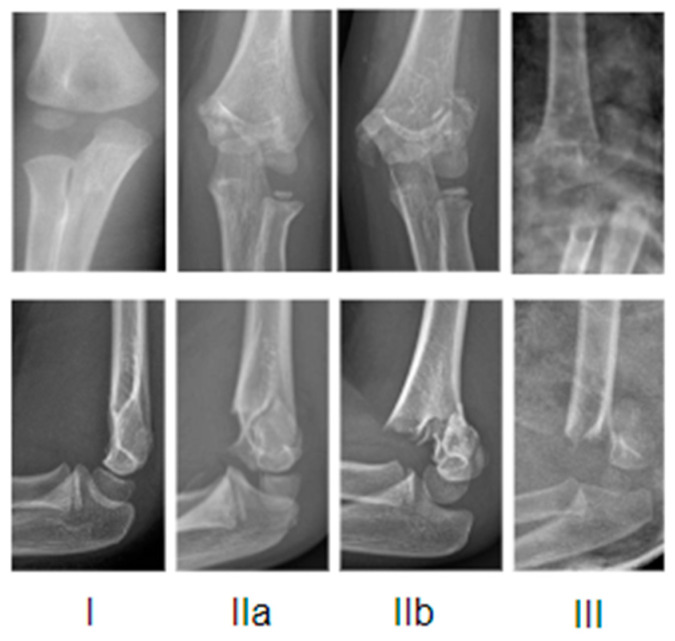
Exemplificative X-rays representation of Gartland classification.

The radiographic Gartland’s classification modified by Wilkins is the most commonly used to describe the fracture pattern: I, IIa, IIb, and III (Figure 1). 

In type I fractures, the fracture line is not easily identifiable and the sign of fat pads can help in making a diagnosis; it may be an occult fracture and the signs of healing could become visible at radiographic follow-up. In type II fractures, the integrity of the posterior cortex is maintained by a thick intact periosteum, but there is angulation of the bone fragments and the anterior humeral line falls anterior to the middle third of the capitulum (in type IIa, there is angulation without rotation; in type IIb, there is rotational and/or translational displacement). Type III fractures are complete and displaced; the direction of displacement is often postero-medial, more rarely postero-lateral. 

There are some radiographic parameters that can be assessed. On the antero-posterior projection it is possible to calculate the Baumann angle (BA), the carrying angle, the humero-ulnar angle and the metaphyseal diaphyseal angle. In lateral projection, the coronoid line, the radio-capitellar line and the anterior humeral line (AHL) can be observed.

The Baumann angle and the Anterior Humera Line are very often used in the literature and in clinical practice [10]. Regarding the BA, type I variation is the one originally described by the author and is the most used, involving measurement of the angle between the axis of the humeral diaphysis and the line parallel to the fossa of the lateral condyle. The resulting angle has a normal value of 75–80° [11]. 

The AHL should intersect the middle third of the capitulum nucleus, giving an idea of the translation of the distal fragment in the sagittal plane after fracture reduction [12]. Rogers et al. [13] first described the AHL by establishing that it should pass through the middle third of the capitulum. Herman et al. [14] argued that the AHL line could pass through either the middle third or the anterior third indifferently in children under 4 years of age, and through the middle third in older children. 

Concerning the treatment, Gartland and Wilkins Type I fractures are stable, with minimal risk of displacement or angulation. The anterior humeral line is preserved and displacement is less than 2 mm. Treatment is non-surgical and represented by immobilization for precautionary antalgic purposes in brachio-metacarpal cast braces with elbow flexion at 60–90°. In type IIa according to Gartland and Wilkins, without a rotational deformity, a coronal malalignment, or a dislocation in extension of the distal fragment, treatment can be conservative with plaster. Stabilization by percutaneous pinning should be considered in Type IIa fractures in which flexion greater than 90° is required to maintain reduction, and in Type IIb fractures. In Type IIb and Type III, the most appropriate timing to ensure safe and reliable synthesis is still debated in the literature [15]. 

The aims of this study were to evaluate the inter- and intra-observer reliability of Gartland’s classification modified by Wilkins and those of the Baumann angle (BA) and of the Anterior Humeral Line (AHL).

## 2. Materials and Methods 

This retrospective single-center observational study was carried out at the Bambino Gesù Children’s Hospital in Rome. The study was conducted in accordance with the Declaration of Helsinki and a notification was sent to the ethics committee of the Hospital. 

We collected the clinical data of 217 consecutive patients with a diagnosis of supracondylar fracture type Gartland and Wilkins IIa, IIb or III, all treated by the same team between 2016 and 2017. Patients with type I supracondylar fractures, those whose clinical radiographic documentation was incomplete or radiographic images were not suitable for the calculation of the chosen parameters were excluded. The medical history and imaging documentation of all patients were collected. 

Four observers participated in the study: Observer 1 (O1—medical doctor at the 5th year of residency in Orthopaedics and Traumatology); Observer 2 (O2—junior grade Orthopaedist with 1 year of experience); Observer 3 (O3—senior Orthopaedist with 15 years of experience); Observer 4 (O4—middle grade Orthopaedist with 5 years of experience). Two evaluations were performed in two different moments (two months apart from the first observation to the second) on the pre-operative radiographs grading the fractures according to the Gartland and Wilkins classification (IIa, IIb, III). In addition, in the immediate postoperative period, the following parameters were calculated by all observers two times (two months apart from the first observation to the second): on the antero-posterior projection, the Baumann angle; on the latero-lateral projection, the Anterior Humeral Line. 

Regarding the AHL, we previously identified seven radiographic areas of the elbow on the lateral projection: zone 0 (AHL that fell out of the anterior humeral cortex), zone 1 (AHL tangent to the anterior humeral cortex), zone 2 (AHL that fell in the anterior third of the capitulum), zone 3 (AHL that fell in the middle third of the capitulum), zone 4 (AHL that fell in the posterior third of the capitulum), zone 5 (AHL tangent to the posterior humeral cortex) and zone 6 (AHL out of the posterior cortex). 

Statistical analysis was performed using the STATA system (Stata, College Station, TX, USA). A *p*-value of less than 0.05 was considered significant. 

For assessing the inter-observer reliability of the Gartland classification, we obtained the kappa measure for each 1 vs. 1 operator; for the intra-observer reliability a Cohen’s kappa was used to examine the results of each operator. 

Regarding the Baumann angle, we calculated the population mean for all the measurements; then, we used the Intraclass Correlation Coefficient (ICC) to assess the inter-observer and the intra-observer reliability. 

The anterior humeral line was analyzed in a double manner: considering it as a discrete variable, the inter-observer reliability was obtained through the kappa measure for each 1 vs. 1 operator; for the intra-observer reliability a Cohen’s kappa was used. Considering the AHL as a continuous variable, both the inter- and the inter-observer reliability were calculated using the ICC.

## 3. Results

The initial population included 217 patients. Of these, 30 were excluded because the clinical radiographic documentation was incomplete and one because the Baumann angle was incalculable due to the closure of the growth nuclei. The sample under study therefore consisted of 186 individuals. It comprised 109 (58.60%) male and 77 (41.39%) female patients. The mean age was 6.5 years with a minimum of 1.2 years and a maximum of 7.7 years. The affected side was the left in 116 (62.36%) patients; the right in 70 (37.6%) patients. In the summer months (in order: August, June, July, September), there was the seasonal peak of fractures, with a clear reduction in the autumn and winter months.

With regard to the traumatic event, in our study the fall occurred mainly during sports activities (16.1%), at home (13.4%), at playground (10.2%), falls from little height e.g., furniture or trees or statues (10.2%), at school (5.4%), in the street (5.4%), in a public park (4.3%), on inflatables (2.7%), from a low wall (2.1%), on trampolines (1.6%), due to road traffic (1.1%), at the beach (1.1%), in osteogenesis imperfecta (0.5%), or due to unknown cause (18.3%). There were four exposed fractures (2.15%) and one elbow dislocation (0.5%); an ipsilateral distal radius fracture was associated with a supracondylar humerus fracture in four patients (2.15%), in one of whom the distal radius was fractured bilaterally. 

Acute vascular deficits were present in four patients (2.15%): in three patients, in the form of a weak radial pulse with immediate resumption of a valid pulse after placement of trans-olecranon traction in the emergency department; in one patient, there was pulselessness at onset due to lesion and thrombosis of the humeral artery which needed exploration and suture in the emergency room, once closed reduction and percutaneous fracture synthesis had been performed. One patient (0.5%) reported major skin complications such as circumferential blisters requiring drainage and renewal of the plaster cast during hospital stay. Eleven patients (5.9%) reported neurological complications (three median nerve deficit, one radial nerve deficit, six anterior interosseous nerve deficit NIA and one unspecified). Concerning the NIA deficit, in one patient the recovery occurred immediately after traction placement, in four patients the improvement was almost complete two months after the trauma and in one patient the improvement was perceived by 5 months post trauma. The patient with radial deficit was lost at follow-up, and the patient with nerve deficit of an unspecified type in the history had almost complete improvement two months after the trauma.

The measurements were performed on 186 subjects by four observers (O1, O2, O3, O4) on two different occasions (a total of 1488 observations). 

### 3.1. Gartland

Regarding the Gartland classification, the amount of inter-observer agreement was higher than the expected one, indicating that we can reject (*p* < 0.05) the hypothesis that the determinations were done randomly. In the first visit, there was agreement among all four observers on 31.2% for type III, 14% for type IIa, and 9.7% for type IIb, with a kappa of 0.73 for type III (substantial), 0.65 for type IIa (substantial), and 0.43 (moderate) for type IIb. The combined K for all the types was 0.61 (substantial). In the second data collection, there was agreement among all four observers on 38.2% for type III, 10.2% for type IIa and 2.7% for type IIb, with a K of 0.61 (substantial) for type IIa e III; and K of 0.26 (fair) for type IIb. Values less than 0.00 indicate poor reliability; 0.00 to 0.20, slight reliability; 0.21 to 0.40, fair reliability; 0.41 to 0.60, moderate reliability; 0.61 to 0.80, substantial agreement; and 0.81 to 1.00, excellent or almost perfect agreement. The results are listed in Table 1. The intra-observer reliability was measured by using the unweighted Cohen’s kappa: O1 79.03% (*p*-value < 0.0001); O2 66.67% (*p*-value < 0.0001); O3 98.92% (*p*-value < 0.0001); O4 81.18% (*p*-value < 0.0001). 

### 3.2. Baumann Angle

The Baumann angle on 186 subjects was evaluated by four observers on two different occasions (a total of 1488 observations). The mean value of the measurements obtained in the first data collection (a total of 744 observations) was 73.5 ± 6.85 with an overall Inter-observer ICC of 0.74 (95% confidence interval = 0.67–0.80), interpreted as “good”. The 744 s visit measurements showed the following population mean: 72.9 ± 6.83, with an inter-observer ICC of 0.77, interpreted as “good” (95% confidence interval = 0.71–0.82). Reliability was considered to be almost perfect for an ICC value of 0.81 to 1.0, good for 0.61 to 0.80, moderate for 0.41 to 0.60, fair for 0.21 to 0.40, and poor for 0.0 to 0.20. The intra-observer reliability between visits was estimated for each operator (O1 ICC = 0.90, 95% confidence interval = 0.87–0.93; O2 ICC = 0.96, 95% confidence interval = 0.94–0.97; O3 ICC = 0.94, 95% confidence interval = 0.92–0.96; O4 ICC = 0.91, 95% confidence interval = 0.87–0.93). The results are listed in Table 2 and Table 3. 

### 3.3. Anterior Humeral Line

Regarding the AHL, the amount of agreement indicates that we can reject (*p* < 0.05) the hypothesis that the determinations were done randomly. 

Interobserver reliability was, for the first visit, 8.6% for AHL that fell in the anterior third of the capitulum, 5.9% for AHL on the middle third and 1.6% for AHL tangent to the anterior humeral cortex; all the other identified areas (AHL that fell out of the anterior humeral cortex, in the posterior third of the capitulum, tangent to the posterior humeral cortex, and out of the posterior cortex) had 0%. For the second visit, there was agreement of classification among all four observers only for 10.8% of AHL that fell in the anterior third and 8.6% in the middle third. Results are listed in Table 4 and Table 5. 

Intraobserver reliability was measured by using the unweighted Cohen’s kappa (interpretation: O3 86.02% > O2 64.52% > O1 56.45%> O4 30.11%). The results are listed in Table 6. 

However, if the variable AHL location category is treated as continuous, Intraclass Correlation coefficient (ICC) is calculated to assess inter-observer reliability and test–retest reliability. Overall, inter-observer ICC of AHL was 0.87 (95% confidence interval = 0.83–0.89), interpreted as “almost perfect”, and 0.80 (95% confidence interval = 0.75–0.84), interpreted as “good”, for the second visit. The intra-observer reliability between visits was estimated for each operator: O1 ICC = 0.85 (95% confidence interval = 0.79–0.88); O2 ICC = 0.66 (95% confidence interval = 0.54–0.74); O3 ICC = 0.91 (95% confidence interval = 0.88–0.93); O4 ICC = 0.85 (95% confidence interval = 0.80–0.89). Results are listed in Table 7 and Table 8.

## 4. Discussion

The mean age of 6.5 years in the study sample is slightly higher than in the literature: 5 years [16], 5.5 years [17], 6 years [18], and 6.2 years [19]. The gender predominance in our study was male. The latter finding is in agreement with the study by Mitchelson et al. [20] and that of Barr. Other studies report no significant differences in the distribution of supracondylar fractures by sex [21]. In our study, the most affected side was left, in agreement with Morrey. 

Regarding the seasonal distribution, in our study the highest number of fractures was observed during the summer. This is in agreement with the study by Holt et al. [17] and the study by Houshian et al. [22]. Farnsworth et al., in California, reported no differences in seasonal distribution, perhaps because the climate there is temperate year-round, compared to the USA and Denmark [23]. Our results are similar to those of Mitchelson and Aparicio regarding the rate of falls during sporting activity (16.1%) and with Barr regarding falls from furniture (10.2%). Holt et al. and Sinikumpu et al. reported the highest incidence of falls from playground equipment (25–40% and 51.5% respectively) [24]. We found the lowest agreement regarding falls from trampolines (1.6%), which is the highest in the literature (Mitchelson 7.3%). 

The overall Inter-observer reliability with regard to the Gartland grades showed the greatest agreement for grade III (first visit, K = 0.73; second visit, K = 0.61; agreement among all observers 31.2% on the first visit and 38.2% on the second visit), then for grade IIa (first visit, K = 0.65; second visit, K = 0.61; agreement among all observers 14% on the first visit and 10.2% on the second visit) and finally grade IIb (first visit, K = 0.43; second visit, K = 0.26; agreement among all observers was 9.7% on the first visit and 2.7% on the second visit). These values are in line with the literature: Barton et al. obtained an overall K-value between 0.59 and 0.77 for each time the observers had performed the radiographic observation [25]; Silveira Rocha obtained an overall inter-observer correlation of K = 0.756 globally [26]; Teo et al. reported a weighted K between 0.66 and 0.68 in their two groups of observers from different geographical locations [27], similarly to Heal et al. [28]. We are also in agreement with Mallo et al., who found a low to moderate agreement for type II and good to excellent for type III [29]. 

The mean value of the Baumann angle in the first visit was 73.5 ± 6.85 (with an overall inter-observer ICC of 0.74 (95% confidence interval = 0.67–0.80), interpreted as “good”); in the second visit, 72.9 ± 6.83 (with an inter-observer ICC of 0.77, interpreted as “good”, 95% confidence interval = 0.71–0.82). These values are in agreement with the literature: Shank et al. found a mean BA of 71.5° with interobserver reliability 0.86 [30]; Silva et al. found r = 0.78, considering a difference of up to 7° in the acceptable error range; Suangyanon et al. found interobserver reliability with r= 0.843 [31]. Several factors influence the measurement of the Baumann angle: the age of the patient and therefore the disappearance of the capitulum cartilage when ossified; internal or external rotation which would result in 5° of variation for the BA for every 10° of rotation, according to Camp et al. [32]; according to Segal et al., the BA value only changes when the intra-rotation varies by −70° and the extra-rotation reaches +40° [33]. The concordance on the BA measurement increases significantly when the visible humerus is >7 cm (*p* = 0.04) [34]. 

Regarding the AHL, overall inter-observer ICC of AHL was 0.87 (95% confidence interval = 0.83–0.89), interpreted as “almost perfect”, and 0.80 (95% confidence interval = 0.75–0.84) for the second visit. The intra-observer reliability between visits was estimated for each operator (O1 ICC = 0.85, 95% confidence interval = 0.79–0.88; O2 ICC = 0.66, 95% confidence interval = 0.54–0.74; O3 ICC = 0.91, 95% confidence interval = 0.88–0.93; O4 ICC = 0.85, 95% confidence interval = 0.80–0.89). Interobserver reliability was, for the first data collection, 8.6% for AHL that intersected the anterior third of the capitulum, 5.9% when AHL fell in the middle third of the capitulum and 1.6% for AHL tangent to the anterior humeral cortex; for all the other identified areas (AHL that fell out of the anterior humeral cortex, in the posterior third of the capitulum, tangent to the posterior humeral cortex and out of the posterior cortex) it was 0%. For the second data collection, there was agreement of classification among all four observers only on 10.8% for AHL in the anterior third of the capitulum and 8.6% in the middle third of the capitulum. The line fell for all observers most frequently in the anterior or middle third of the capitulum nucleus. More rarely, the AHL fell out of the nucleus posteriorly, so the fractures were rarely hypercorrected. However, if AHL is treated as a continuous variable, our ICC value is similar to that obtained by Shimizu et al. (0.75) [35]. The quality of the radiographic image, the intra rotation or external rotation of the humerus, and the size of the capitulum augmentation nucleus are the influential factors on the AHL measurement.

A computer-aided evaluation of the radiographic images should be considered to improve the variability of measurements and to detect and classify the fractures. 

For complex articular fracture patterns, a computed tomography (CT) with 3D reconstruction could be helpful in preoperative evaluation and accurate reduction planning [36,37]. Some authors presented good results after using a virtual reality-based simulator for training the reduction of the fracture [38]. Also, the use of artificial intelligence (AI) has been used for fracture detection, but its algorithms must be better tested [39].

In our study, the reported acute vascular deficits were present in four patients (2.15%), while nerve deficits were present in 5.9% of the cases, which is lower than in the literature. 

## 5. Conclusions

Gartland’s classification modified by Wilkins has substantial reliability and reproducibility. The overall inter-observer reliability with regard to the Gartland grades showed the greatest agreement for grade III, then for grade IIa and finally grade IIb. 

The latter were the most difficult to evaluate due to their intrinsic features. Indeed, the evaluation of the translation and rotation of the fracture’s fragments on a bidimensional radiographic image could be difficult.

Baumann angle and AHL have a high degree of reliability and give an idea of the quality of the reduction achieved.

Given the above, the application of modern detection software and computer aided diagnosis should be considered in order to overcome the variability in inter-observer measurements.

The present study appears to be the first to have simultaneously assessed the interobserver reliability of Gartland’s classification modified by Wilkins, that of Baumann’s angle and that of the Anterior Humeral Line in a considerable sample of patients treated in only two years at a single hospital center. 

On the basis of the results achieved, the following limitations of the study can be inferred: (i) the lack of a long clinical radiographic follow-up and (ii) the absence of a specific software for the measurements of radiographic parameters. 

Further studies will be necessary to assess the distant outcomes in these patients and to better understand how to minimize inter-observer differences in evaluation.

## Figures and Tables

**Table 1:** Inter-observer reliability of Gartland Classification.

Gartland Type	Evaluation	*K*	*Z*	*p*-Value	Interpretation
IIa	1° evaluation	0.65	21.87	<0.0001	substantial
	2° evaluation	0.61	20.58	<0.0001	substantial
IIb	1° evaluation	0.43	14.49	<0.0001	moderate
	2° evaluation	0.26	8.86	<0.0001	fair
III	1° evaluation	0.73	24.39	<0.0001	substantial
	2° evaluation	0.61	20.38	<0.0001	substantial

*K*: kappa measure, *Z*: zeta score.

**Table 2:** Inter-observer reliability of Baumann angle.

Evaluation	ICC	*p*-Value	Interpretation
1° evaluation	0.74	<0.001	Good
2° evaluation	0.77	<0.001	Good

**Table 3:** Intra-observer-reliability of Baumann angle.

Observer	ICC	*p*-Value	Interpretation
O1	0.90	<0.001	Almost perfect
O2	0.96	<0.001	Almost perfect
O3	0.94	<0.001	Almost perfect
O4	0.91	<0.001	Almost perfect

**Table 4:** Inter-observer reliability AHL as discrete variable in the first visit.

Zone *	Agreement	*K*	*Z*	*p*-Value
0	0%	0.43	14.62	<0.0001
1	1.6%	0.31	10.58	<0.0001
2	8.6%	0.18	6.19	<0.0001
3	5.9%	0.17	5.78	<0.0001
4	0%	0.17	5.97	<0.0001
5	0%	0.21	7.21	<0.0001
6	0%	−0.0013	-0.04	0.5179

* Zones are listed in Materials and Methods section.

**Table 5:** Inter-observer reliability AHL as discrete variable in the second visit.

Zone *	Agreement	*K*	*Z*	*p*-Value
0	0%	0.08	2.89	0.0019
1	0%	0.15	5.32	<0.0001
2	10.8%	0.20	6.94	<0.0001
3	8.6%	0.29	9.91	<0.0001
4	0%	0.00	0.22	0.4124
5	0%	0.26	8.74	<0.0001
6	0%	NA	NA	NA

* Zones are listed in Materials and Methods section.

**Table 6:** Intra-observer reliability of AHL as discrete variable.

Observer	Agreement	*K*	*Z*	*p*-Value
O1	56.45%	0.38	9.04	<0.0001
O2	64.52%	0.37	6.41	<0.0001
O3	86.02%	0.73	13.99	<0.0001
O4	30.11%	0.17	7.02	<0.0001

**Table 7:** Inter-observer reliability of AHL as a continuous variable.

Evaluation	ICC	*p*-Value	Interpretation
1° evaluation	0.87	<0.001	Almost perfect
2° evaluation	0.80	<0.001	Good

**Table 8:** Intra-observer reliability of AHL as a continuous variable.

Observer	ICC	*p*-Value	Interpretation
O1	0.85	<0.001	Almost perfect
O2	0.66	<0.001	Good
O3	0.91	<0.001	Almost perfect
O4	0.85	<0.001	Almost perfect

## Data Availability

Datasets generated and/or analyzed during the current study are available from the corresponding author upon reasonable request.
